# Ex Vivo Computed Tomographic Morphometry and Motion of the Native and Fractured Equine Accessory Carpal Bone

**DOI:** 10.3390/ani16081132

**Published:** 2026-04-08

**Authors:** Jennifer Gernhardt, Thomas Reuter, Guido Fritsch, Nicole Schulze, Kathrin Mählmann, Christoph Lischer

**Affiliations:** 1 Equine Clinic, Freie Universität Berlin, Oertzenweg 19b, 14163 Berlin, Germany; guhl.nicole@gmail.com (N.S.); kathrin.maehlmann@fu-berlin.de (K.M.); christoph.lischer@fu-berlin.de (C.L.); 2 ICM—Institut Chemnitzer Maschinen- Und Anlagenbau e.V., 09117 Chemnitz, Germany; t.reuter@icm-chemnitz.de; 3 Leibniz Institute for Zoo and Wildlife Research, Forschungsverbund Berlin e.V., 10315 Berlin, Germany; fritsch@izw-berlin.de

**Keywords:** accessory carpal bone, equine carpus, dynamic computed tomography, bone morphometry, carpal kinematics

## Abstract

The accessory carpal bone (ACB) is an important component of the equine carpus and a clinically relevant site of fracture, yet its detailed morphology and motion characteristics remain incompletely characterized. The objectives of this ex vivo study were to characterize the three-dimensional morphometry of the native ACB using computed tomography (CT), to assess its kinematics during carpal flexion and extension using dynamic 3D CT, and to evaluate fragment behavior following a standardized vertical fracture. Twelve equine forelimbs underwent CT imaging in the neutral carpal position, maximal flexion, and maximal extension before and after standardized osteotomy simulating a vertical plane fracture. Morphometric measurements included dorsopalmar width, lateromedial thickness, proximodistal length, and radii of medial concavity and lateral convexity. The ACB exhibited consistent geometry among specimens of comparable size. Dynamic imaging revealed independent physiological motion relative to the other carpal bones of the ACB, characterized by medial movement of its medial concave surface toward the caudal aspect of the radius during flexion. Following fracture creation, widening of the fracture gap and proximal displacement of the palmar fragment occurred predominantly in flexion, whereas the neutral carpal position and maximal extension promoted fracture gap closure and distal displacement. These findings improve understanding of ACB morphology and biomechanics and may have implications for fracture pathogenesis and surgical fixation strategies.

## Simple Summary

Fractures of the accessory carpal bone are an important cause of lameness in horses and can severely affect athletic performance. Despite their clinical relevance, little is known about the exact shape of this bone and how it moves during flexion and extension of the carpus. A better understanding of these factors may help explain how fractures occur and improve surgical treatment. In this study, computed tomography was used to examine the accessory carpal bone in equine forelimbs and to observe its motion during controlled flexion and extension of the joint. In addition, fractures were experimentally created to investigate how bone fragments move in different limb positions. The results showed that the accessory carpal bone does not remain stationary but moves independently during joint flexion toward the caudal aspect of the radius. The study also demonstrated that the position of the limb influences whether the fracture gap opens or closes and how the fractured palmar fragment shifts. Importantly, carpal flexion was found to increase the fracture gap, suggesting that limb flexion should be minimized during initial management. Furthermore, the shape of the bone was found to be consistent among horses of similar size. These findings improve the understanding of fracture development and may help veterinarians choose better surgical techniques and design implants specifically adapted to this bone.

## 1. Introduction

The accessory carpal bone (ACB) forms part of the proximal carpal row and articulates dorsolaterally with the radius and the ulnar carpal bone through its proximal and distal articular surfaces, respectively [1]. Its medial concave surface and distal ligaments contribute to the lateral wall of the carpal canal, while the palmar aspect serves as an attachment for the flexor retinaculum [1,2]. A smooth sulcus on its lateral surface accommodates the tendon of the extensor carpi ulnaris muscle, and the flexor carpi ulnaris muscle inserts on its proximal margin [3]. Functionally, the ACB does not directly bear weight and is often considered a sesamoid bone [1].

The principal motions of the carpus are flexion and extension. Movement is initiated primarily at the antebrachiocarpal joint, with progressive recruitment of the middle carpal joint as flexion increases [4,5]. The middle carpal joint acts largely as a hinge, whereas the antebrachiocarpal joint allows rotational motion with an additional gliding component [6]. Independent motion of the carpal bones has been well documented: for example, the radial carpal bone translates distally relative to the intermediate and ulnar carpal bones [5]. In contrast, the kinematics of the ACB remain poorly characterized. It has been suggested that the accessory carpal bone does not move during flexion. However, this assumption is not supported by quantitative data.

**Figure 1:**
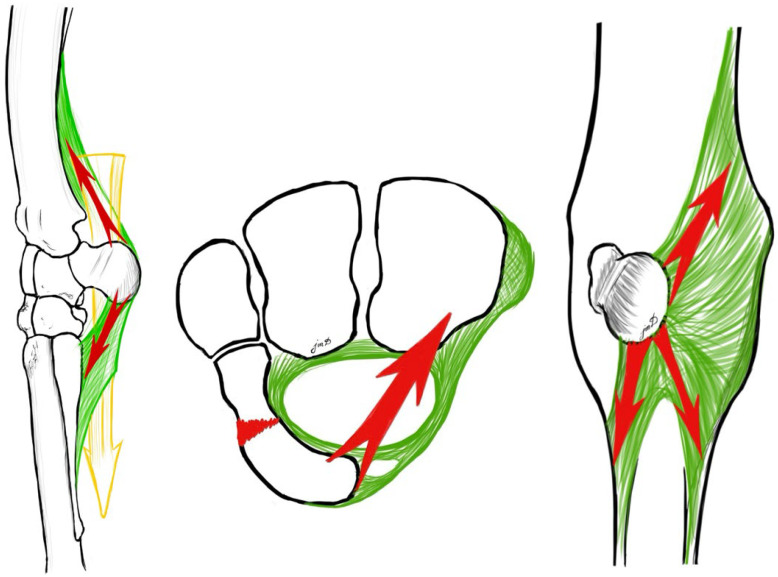
**Left**: Lateral view of the left carpus in hyperextension (dorsal to the left). Illustration by Prof. Jean-Marie Denoix demonstrating opposing tensile forces acting on the accessory carpal bone (ACB). Ligamentous attachments and the antebrachial fascia generate proximally and distally directed traction forces (red arrows). In addition, the flexor tendons within the carpal canal are subjected to increased tension during hyperextension of the limb. **Middle**: Transverse view of the proximal carpal row (dorsal to the top). During hyperextension, the flexor retinaculum is maximally tensioned, exerting compressive force on the palmar aspect of the ACB while simultaneously applying a medially directed force (red arrow). **Right**: Tensile forces exerted by the antebrachial fascia (red arrows) in distal and proximomedial directions [16].

Fractures of the accessory carpal bone are most commonly oriented in the vertical plane (also referred to as frontal plane fractures in the literature) [7,8,9,10,11,12]. Clinically, falls onto a flexed carpus have been proposed as a common mechanism for ACB fractures [8,13,14,15], supporting the “nutcracker” hypothesis in which the bone is compressed between the radius and the third metacarpal bone [7]. Alternatively, fracture development has been attributed to hyperextension of the carpus [16] (Figure 1).

Management of ACB fractures ranges from conservative treatment to surgical stabilization. Several surgical techniques and implants have been described, including lag screw fixation and plate osteosynthesis [12,14,17,18,19]. To the authors’ knowledge, the motion of the ACB has not yet been investigated, and anatomical reference data that could guide implant design are lacking. Moreover, optimal plate placement requires, among other factors, an understanding of the ACB’s tension side, which is influenced by its kinematics in both the intact and fractured states.

The objectives of this study were to (1) describe the morphometry of the ACB, (2) characterize its motion using dynamic three-dimensional computed tomography (3D CT), and (3) determine whether a vertical plane fracture alters its kinematics. It was hypothesized that the intact ACB exhibits independent physiological motion relative to the other carpal bones during carpal flexion and that creation of a vertical fracture configuration results in displacement of the palmar fragment.

## 2. Materials and Methods

### 2.1. Collection of Specimens

Twelve cadaveric thoracic limbs were obtained from horses euthanized for reasons unrelated to carpal joint pathology. The limbs were transected at the cubital joint, sealed in plastic bags in an extended position, and stored at −20 °C. In the slaughtered limb, the skin had been removed, whereas the soft tissues of the remaining limbs were intact. Prior to experimentation, all limbs were thawed at room temperature for 24 h.

These cadaveric limbs were used for dynamic CT imaging to assess range of motion, as well as for experimental fracture creation.

Additionally, CT datasets from twelve forelimbs of nine adult horses were retrospectively included for morphometric analysis. These horses had undergone CT between January 2024 and September 2025 for reasons unrelated to ACB pathology.

### 2.2. Testing Device

**Figure 2:**
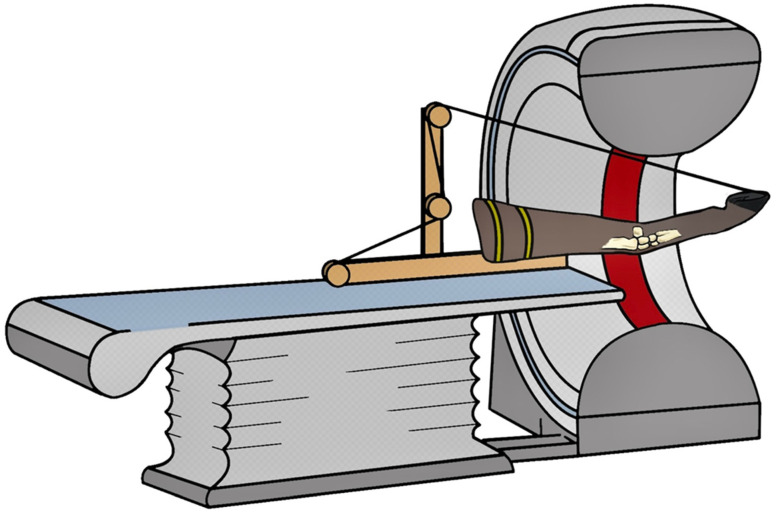
Custom-designed motorized testing device for dynamic computed tomographic (CT) scanning (320-detector-row CT scanner, Aquilion One, Canon Medical Systems) [21]. The radius was positioned horizontally with the caudal aspect facing upward and secured to the custom-made apparatus (yellow straps), which consisted of a wooden base plate, frame, and rod. The carpus was centered within the CT field of view (indicated in dark red). The rod and base plate incorporated pulleys to enable controlled flexion and extension of the carpus.

A custom-designed motorized testing device was used to position and manipulate the limb specimens during dynamic CT scanning [20]. The radius was fixed horizontally with the caudal aspect facing upward in an apparatus consisting of a wooden base plate, frame, and rod. The rod and base plate incorporated pulleys to permit controlled flexion and extension of the carpus. For this purpose, a rope was secured to a predrilled hole in the dorsal aspect of the weight-bearing border of the hoof wall (Figure 2).

A single rope was used for both flexion and extension. For carpal flexion, the rope was tensioned on the palmar side of the limb. During CT scanning, linear motion inducing alternating carpal flexion was generated by a remote-controlled multiphase motor operated at 25 V and 3.3 A. A linear displacement of 0.8 m was completed within 15 s, corresponding to a mean velocity of 0.053 m/s. Motor parameters were kept constant throughout all experimental trials. For extension, the same rope was repositioned to the dorsal side of the limb prior to each cycle and tightened maximally using the motor.

### 2.3. Computed Tomographic Imaging

Cadaveric limbs were scanned in intermittent sequential mode using a 320–detector-row CT scanner (Aquilion One; Canon Medical Systems, Zoetermeer, The Netherlands) with the following parameters: 512 × 512 pixel field of view, 0.5 mm slice thickness, and 0.35 s tube rotation time at 100 kVp and 280 mAs (100 effective mAs).

Three scan positions were obtained: the neutral carpal position, maximal flexion, and maximal extension. The neutral carpal position was defined as the initial resting position of the limb within the testing device prior to the onset of motorized flexion and was standardized across specimens by consistent positioning within the experimental setup. Eight limbs were scanned prior to fracture creation, and all twelve limbs were scanned after fracture creation.

Computed tomographic examinations of cases identified from the medical records were performed using a 32-slice scanner (Canon Medical Systems, Amstelveen, The Netherlands) with a 512 × 512 pixel field of view, 135 kV, 370 mA, and a 1 mm slice thickness.

### 2.4. Fracture Creation

**Figure 3:**
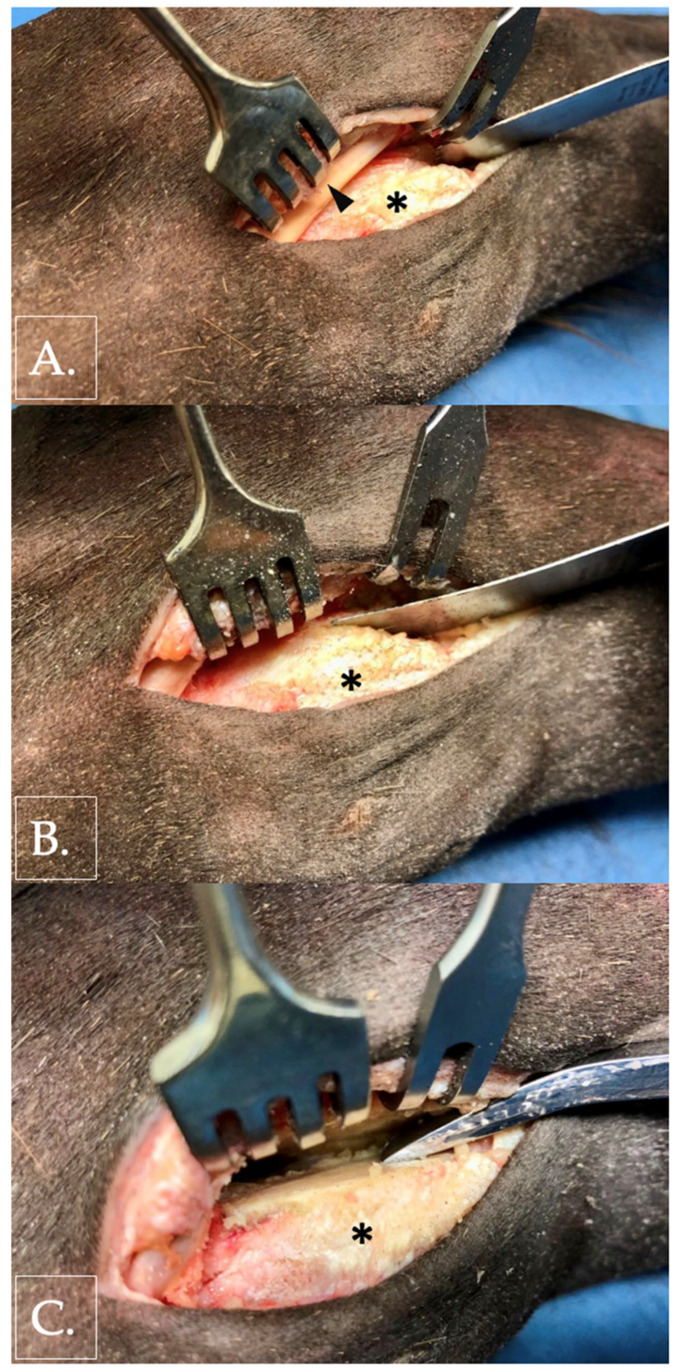
Photographic documentation of artificial fracture creation in a right forelimb (lateral aspect). Radius positioned to the left, metacarpus to the right, and dorsal oriented to the top. The ACB is marked with an asterisk. (**A**) Oscillating saw positioned palmar to the tendon of the ulnaris lateralis muscle (arrowhead). (**B**) Creation of a vertical plane fracture. (**C**) Confirmation of complete osseous separation by insertion of a periosteal elevator into the fracture gap.

Vertical plane fractures were created using an oscillating saw. The ACB was approached through an approximately 5 cm longitudinal skin incision placed over the tendon of the extensor carpi ulnaris muscle (Figure 3). The tendon sheath of the extensor carpi ulnaris was incised to permit mobilization of the tendon.

A standardized osteotomy was performed palmar to the extensor sulcus using an oscillating saw (Colibri II; DePuy Synthes, Johnson & Johnson MedTech, New Brunswick, NJ, USA) with a 50 mm × 14 mm saw blade (cut thickness 0.6 mm; DePuy Synthes, Johnson & Johnson MedTech, USA). This location reproduced the vertical plane fracture configuration most frequently reported in the literature. Complete separation of the bone was confirmed by inserting a periosteal elevator into the osteotomy gap and mobilizing the palmar fragment [22].

Skin incisions were closed using a simple continuous suture pattern (Prolene^®^ USP 0; Ethicon, Johnson & Johnson MedTech, New Brunswick, NJ, USA) to minimize gas accumulation during CT imaging.

### 2.5. Morphometric Measurements

To quantify the anatomical dimensions of the ACB, measurements were obtained from CT datasets of eight cadaveric limbs and from retrospective clinical CT datasets using open-source software (Horos, version 4.0.1; Horos Project, Annapolis, MD, USA). Topographic measurements were performed in a total of twenty limbs (eight cadaveric specimens and twelve limbs obtained from clinical records).

Multiplanar reconstructions (MPRs) were aligned relative to the longitudinal axis of the radius, defining the sagittal, transverse and dorsal planes. In this orientation, the reference cross was positioned at three defined reference points:

Point R (proximal articular surface of the ACB with the caudal radius) was defined as the geometric center of the proximal articular surface.

Point U (distal articular surface of the ACB with the ulnar carpal bone) was defined as the geometric center of the distal articular surface.

Point C (midpoint between both articular surfaces of the ACB) was defined as the midpoint of the proximodistal distance between the proximal articular margin of the ACB with the radius and the distal articular margin with the ulnar carpal bone.

**Figure 4:**
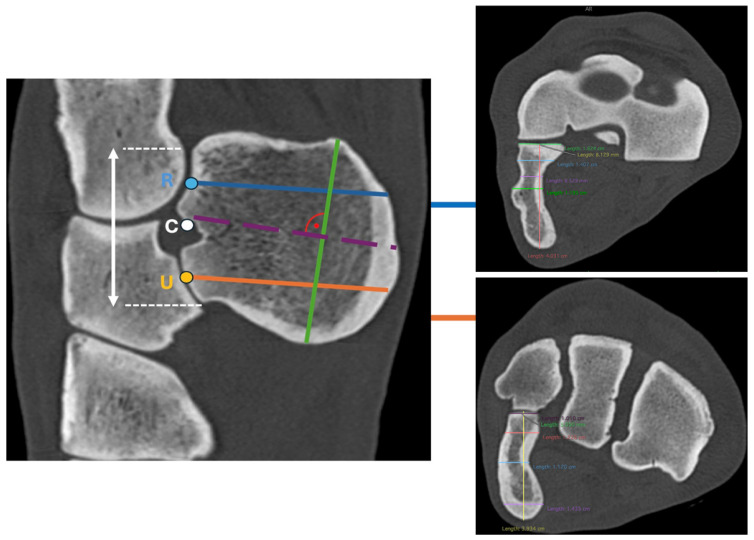
**Left**: MPR CT image in the sagittal plane showing the radius, ulnar carpal bone, fourth carpal bone, and ACB. Dorsal is oriented to the left. Blue line: Measurement at point R (center of the proximal ACB–radius articulation), perpendicular to the articular surface of the ACB. Orange line: Measurement at point U (center of the distal ACB–ulnar carpal bone articulation), perpendicular to the articular surface of the ACB. Purple dashed line: Reference point C (midpoint between the proximal and distal ACB articular surfaces), used to assess medial concavity and lateral convexity. Green line: Proximodistal length of the ACB measured along the longitudinal axis of the bone. **Right**: Transverse plane MPR CT images illustrating measurement example of dorsopalmar width and lateromedial thickness at point R (**top**) and point U (**bottom**). Dorsal is oriented to the top.

To measure the dorsopalmar width at points R and U, the MPR was oriented in the transverse plane at the mediolateral midpoint of the respective articular surface of the ACB. The dorsopalmar distance from the articular surface to the palmar margin of the ACB was measured along a line perpendicular to the articular surface of the ACB (Figure 4).

The lateromedial thickness was measured at point R at three predefined locations: the dorsal border of the sulcus for the tendon of the extensor carpi ulnaris muscle, the deepest point of the sulcus, and the palmar border of the sulcus.

The lateromedial thickness was measured at point U at three predefined locations: the dorsal margin of the medial concavity, the deepest point of the concavity, and the palmar margin of the concavity.

At point C, the MPR was aligned with the sagittal axis of the ACB. In this plane, the ACB was visually divided into two equal halves at point C. In the transverse plane, the reconstruction was oriented along the sagittal axis of the ACB, corresponding to the theoretical trajectory of a cortical screw inserted without breaching either the medial or lateral cortex. In this orientation, the proximodistal length of the ACB was measured.

**Figure 5:**
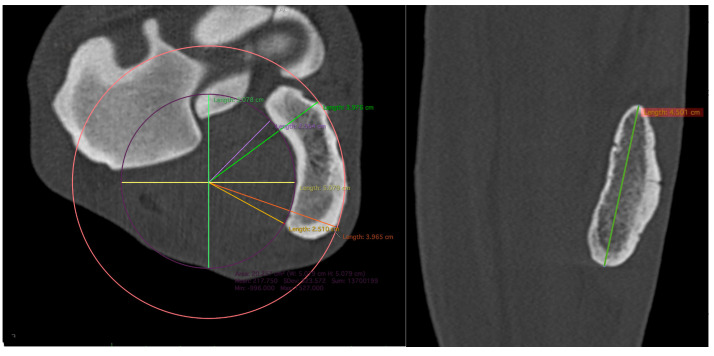
**Left**: Transverse plane MPR CT image illustrating measurement at point C (midpoint between the proximal and distal ACB articular surfaces), demonstrating determination of the radius of the medial concavity and lateral convexity using a best-fit circle. **Right**: Frontal plane MPR CT image illustrating measurement of the proximodistal length of the ACB at point C, which was measured along the longitudinal axis of the bone.

The radius of curvature of the medial concavity was determined by fitting a circle to the medial concave surface of the ACB. A second circle with the same centre was projected onto the lateral convex surface to ensure geometric symmetry. The radius was measured at two locations, namely at the dorsal and palmar cortical margins, and was defined as the linear distance from the common centre to the intersection of the fitted circle with these margins (Figure 5).

### 2.6. Range of Motion of Cadaveric Limbs

The angle between the longitudinal axes of the radius and the third metacarpal bone was measured in maximal flexion and maximal extension. For maximal flexion, the scan at the end of the flexion phase was selected, and the MPR was aligned with the sagittal axes of the radius and the third metacarpal bone. For maximal extension, the corresponding scan was selected, and the MPR was aligned along the sagittal plane defined by the radius, intermediate carpal bone, third carpal bone, and third metacarpal bone. Dynamic 3D reconstructions were generated from CT datasets to visualize ACB motion (Supplementary S1 and S2).

Following fracture creation, two separate assessments were performed: (1) evaluation of interfragmentary distance, with the fracture gap categorized as open or closed, and (2) evaluation of palmar fragment displacement, classified as absent, proximally, or distally displaced. Assessments were based on MPR CT images of all twelve cadaveric limbs obtained in the neutral carpal position, maximal flexion, and maximal extension. The evaluations were conducted independently by four veterinarians: two board-certified surgeons, one experienced surgeon, and one surgical resident.

### 2.7. Statistical Analysis

Descriptive statistics were calculated for all morphometric measurements, joint angles, interfragmentary distance, and palmar fragment displacement.

Each parameter was measured three times within a one-month period by the same observer. Therefore, the measurements are dependent.

The assumption of normality was assessed by visual inspection of histograms and quantile–quantile (Q–Q) plots and further evaluated using the Kolmogorov–Smirnov test (*p* > 0.05). Homogeneity of variances was evaluated using Levene’s test (*p* > 0.05). All variables showed normal distribution and homogeneity of variance and could therefore be analyzed using a *t*-test and one-way ANOVA with repeated measures. When appropriate, paired *t*-tests with Bonferroni-adjusted *p*-values were performed for post hoc comparisons. Sphericity was evaluated using Mauchly’s test, if violated (*p* < 0.05), the Greenhouse–Geisser correction was applied. Statistical significance was set at *p* < 0.05. The agreement between the three measurement runs for the 15 parameters was assessed using Bland–Altman analysis. For each pair of parameters, the mean differences (bias) and the 95% limits of agreement (LoA) were calculated. Scatter plots of the differences against the mean values allowed for visual inspection of systematic deviations [23,24].

The assessment of agreement between evaluators was based on the systematization according to Landis et al. Theoretically, it can range from perfect (1) to poor (0). Agreement was interpreted in increments of 0.2 as follows: 0.00–0.20, poor agreement; 0.21–0.40, fair agreement; 0.41–0.60, moderate agreement; 0.61–0.80, substantial agreement; and 0.81–1.00, almost perfect agreement [19].

Data were recorded in Microsoft Excel (Version 16.96.1; Microsoft Inc., Redmond, WA, USA). Statistical analyses were performed using MATLAB (Version 2025a; The MathWorks Inc., Natick, MA, USA) and RStudio (Version 2026.01.0) with R (Version 4.5.2; R Foundation for Statistical Computing, Vienna, Austria) [25,26,27,28].

## 3. Results

### 3.1. Study Population

The cadaveric sample comprised six horses, including five adults (two Hanoverians, two Trotters, and one Friesian) and one Hanoverian yearling. The cohort included two geldings, two mares, and two stallions. The median age was 16.2 years (IQR: 2.7–17.7), and the median body weight was 500.0 kg (IQR: 370.0–567.5). The age, breed, sex, and body weight of one horse were unknown, as the specimen originated from a slaughterhouse.

The CT datasets were obtained from nine horses and included four Warmbloods, two American Quarter Horses, one German Riding Pony, one Paint Horse, and one Friesian cross. The cohort comprised four geldings, three mares and two stallions. The median age was 12.7 years (IQR 7.8–14.7) and the median body weight was 550.0 kg (IQR 540.0–570.0).

### 3.2. Measurement Repeatability

No significant differences were detected among the three repeated measurements (*p* > 0.05), confirming measurement repeatability. Bland–Altman analysis demonstrated that the mean differences (bias) for all parameters were close to zero, with narrow 95% limits of agreement (LoAs), indicating good reproducibility. No systematic trends or dependencies on measurement magnitude were observed (Supplementary S3).

### 3.3. Morphometric Measurements

Morphometric measurement obtained from CT datasets revealed a mean dorsopalmar width at point R of 3.91 ± 0.37 cm (Supplementary S4). Lateromedial thickness at point R measured 1.43 ± 0.16 cm dorsal to the sulcus, 1.09 ± 0.14 cm palmar to the sulcus, and 0.81 ± 0.11 cm at the deepest point of the sulcus.

At point U, the mean dorsopalmar width was 4.39 ± 0.31 cm. Lateromedial thickness measured 1.43 ± 0.22 cm at the dorsal margin of the medial concavity, 1.33 ± 0.19 cm at its deepest point, and 1.70 ± 0.20 cm at the palmar margin.

The mean proximodistal length measured at reference point C, corresponding to the broadest longitudinal dimension was 4.28 ± 0.32 cm.

At point C, the radius of curvature of the medial concavity measured 2.61 ± 0.52 cm dorsally and 2.59 ± 0.52 cm palmarly. The radius of curvature of the lateral convexity measured 4.12 ± 0.50 cm both dorsally and palmarly.

### 3.4. Carpal Range of Motion and ACB Motion

**Figure 6:**
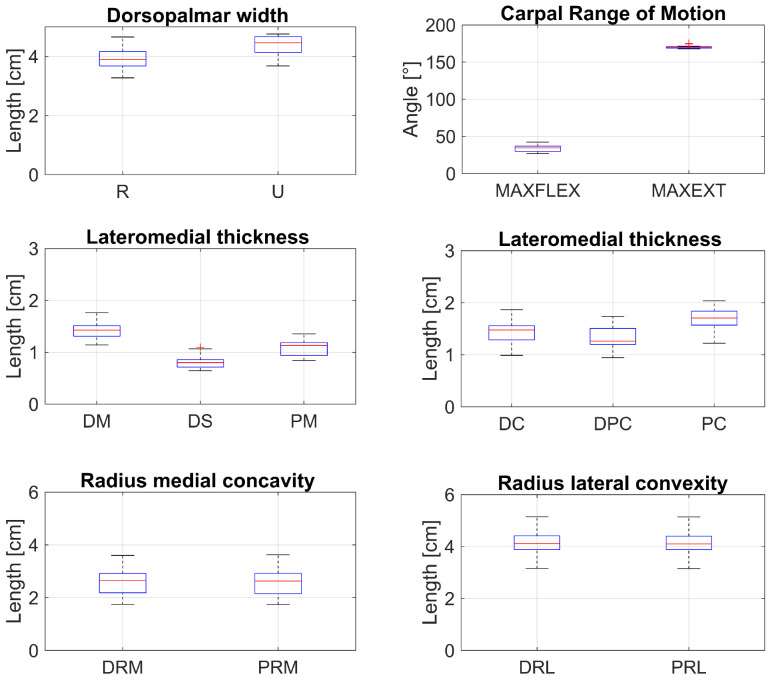
Boxplots showing the distribution of analyzed variables. The central line indicates the median, boxes the interquartile range (IQR), whiskers extend to 1.5 × IQR, and crosses denote outliers. Sample size: n = 60 per variable, except for angle measurements (n = 36 and n = 33). Proximodistal length was excluded from the boxplot because no comparable data were available. Abbreviations: R (center of the proximal ACB–radius articulation), U (center of the distal ACB–ulnar carpal bone articulation), Angle in maximum flexion (MAXFLEX) and maximum extension (MAXEXT); sulcus—dorsal margin (DM), deepest point (DS), palmar margin (PM); medial concavity—dorsal margin (DC), deepest point (DPC), palmar margin (PC); radii of curvature—dorsal radius of medial concavity (DRM), palmar radius of medial concavity (PRM), dorsal radius of lateral convexity (DRL), palmar radius of lateral convexity (PRL).

Maximal flexion angles were available for eight limbs, and maximal extension angles were available for seven limbs. Mean carpal angles were 33.66 ± 4.63° for maximal flexion (n = 12) and 169.99 ± 1.80° for maximal extension (n = 11) (Figure 6).

**Figure 7:**
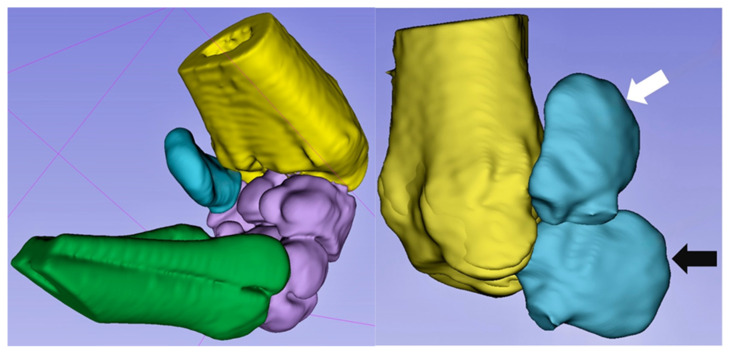
Three-dimensional reconstruction of a flexed carpus generated using 3D Slicer [29,30]. **Left**: Palmaromedial view of the left carpal joint. The radius is shown in yellow, the ACB in blue, the remaining carpal bones in purple, and the metacarpus in green. The medial surface of the ACB moves toward the caudal radius during flexion, illustrating its independent motion. **Right**: Lateral aspect of the left radius and ACB. Dorsal is oriented to the left and the distal radiocarpal joint to the bottom. The carpal bones are removed. During carpal flexion (white arrow), the palmar margin of the ACB demonstrates combined dorsal and medial movement relative to its position in extension (black arrow).

Dynamic CT reconstruction demonstrated independent physiological motion relative to the other carpal bones of the ACB, characterized by medial displacement toward the caudal radius with progressive carpal flexion (Figure 7) (Supplementary S1 and S2). No lateral movement was observed during maximal extension.

### 3.5. Fracture Gap Configuration

Interfragmentary distance varied with limb position. Interobserver agreement was substantial to almost perfect according to the classification of Landis et al. (Fleiss’ kappa = 0.672–1.000) [31].

Minor disagreement between evaluators was observed in a small number of cases. In the neutral carpal position (two specimens) and in maximal extension (one specimen), three evaluators rated the fracture gap as not visible, whereas one evaluator rated it as visible. In maximal flexion, disagreement occurred in one specimen, with three evaluators rating the fracture gap as visible and one as not visible. Two specimens showed a persistent visible fracture gap in both the neutral position and maximal extension. In cases where complete agreement between evaluators was not achieved, the final classification was based on the majority decision (Table 1).

### 3.6. Palmar Fragment Displacement

Palmar fragment displacement also varied with limb position. In the neutral carpal position, ten specimens showed no displacement and two distal displacement. In maximal flexion, eight specimens showed proximal displacement, three no displacement, and one distal displacement. In maximal extension, eight specimens showed distal displacement and four no displacement.

Minor disagreement between evaluators was observed in a small number of cases. In maximal flexion, disagreement occurred in four specimens. In one specimen, two evaluators classified the palmar fragment as proximally displaced and two as not displaced; this case was therefore not assigned to a displacement category. In another specimen, three evaluators classified it as proximally displaced and one as not displaced. In two specimens, one evaluator classified it as proximally displaced, whereas three classified it as not displaced.

In maximal extension, disagreement occurred in one specimen, in which one evaluator classified the fragment as proximally displaced and three as not displaced. When complete agreement was not achieved, the final classification was based on the majority decision (Table 2). No consistent pattern of interobserver disagreement was observed across the different limb positions.

## 4. Discussion

The findings of the present study support the hypothesis that the ACB exhibits independent physiological motion relative to the other carpal bones during carpal flexion. A consistent medial movement of its concave surface toward the caudal aspect of the radius was observed, suggesting that the ACB is not merely a static sesamoid structure but contributes to carpal kinematics. To the authors’ knowledge, this specific motion pattern has not previously been described, and detailed morphometric data for the ACB have been lacking.

Despite its complex three-dimensional architecture, the present data demonstrate that the geometric configuration of the ACB is remarkably consistent among horses of comparable age and body weight across different breeds (Supplementary S5 and S6). This morphological uniformity may facilitate the development of anatomically contoured, size-adapted implants and contribute to more standardized surgical approaches for ACB fracture repair.

Measurements were obtained using consistent anatomical landmarks to ensure reproducibility. The greatest variability was observed in the transverse plane at point C, likely reflecting anatomical differences in cortical morphology rather than inconsistencies in landmark identification. In some specimens, irregular cortical contours limited the precision of the best-fit circle method and required minor adjustments based on visually estimated curvature.

Additional limitations relate to the sagittal orientation of measurements at points R and U. Alignment along the longitudinal axis of the radius and measured along a line perpendicular to the articular surface of the ACB improved reproducibility but may have introduced slight deviations from the true axial plane in some specimens, potentially affecting dorsopalmar length measurements. These compromises were accepted to maintain consistent and reproducible measurement conditions.

A further limitation of this study is the use of different CT scanners and protocols, including varying slice thicknesses. Although the dynamic CT scans were acquired with a smaller slice thickness (0.5 mm), motion during image acquisition reduced the effective image resolution. In contrast, static scans with a slice thickness of 1 mm provided higher image quality. These factors may have influenced the detection and measurement of subtle displacements and small fracture gaps.

A “nutcracker” mechanism involving compression of the ACB between the radius and the third metacarpal bone has been proposed [7]. However, the precise anatomical interfaces responsible for this compression remain incompletely defined.

The present findings suggest that compressive forces may instead occur between the medial surface of the ACB and the caudal radius, particularly near the radial physeal scar (Figure 7), rather than exclusively between the radius and the third metacarpal bone. During carpal flexion, medial displacement of the ACB brings its concave surface closer to the caudal aspect of the radius. However, this interaction alone does not constitute a complete “nutcracker” configuration, as effective compression would require a counteracting force from the third metacarpal bone.

A greater degree of carpal flexion may occur in vivo than was achieved in the experimental setup. Similarly, the degree of carpal extension may also be greater under physiological conditions. To approximate maximal flexion, an additional cadaveric specimen was therefore flexed until the hoof was positioned lateral to the elbow joint and secured before CT imaging (Supplementary S7). In this configuration, the ACB approached both the caudal radius and the head of the fourth metacarpal bone.

Even under this extreme degree of flexion, a measurable gap remained between the ACB and the head of the fourth metacarpal bone. Although force transmission through surrounding soft tissues cannot be excluded, the absence of direct osseous contact suggests that compressive loading between these structures may be limited.

Consequently, the “nutcracker” mechanism as the sole etiopathogenetic explanation appears questionable, and hyperextension remains a plausible alternative. In this context, the accessory carpal bone may be subjected to bending forces under maximal loading conditions. Under maximal carpal loading, tensile forces generated by the multiple ligamentous and muscular attachments of the ACB are counteracted by the flexor retinaculum, producing dorsopalmar compressive forces on the medial aspect of the bone and corresponding tensile forces on the lateral aspect, which may contribute to vertical plane fracture formation [16]. However, this interpretation remains hypothetical and requires further investigation.

As this study was conducted under ex vivo conditions, the influence of muscular forces, particularly contraction of the extensor carpi ulnaris and flexor carpi ulnaris muscles, could not be assessed. Under physiological conditions these forces may further influence ACB motion and joint loading, and the proposed mechanism therefore requires investigation under physiological loading conditions.

The exact location of the (naturally occurring) fracture line within the ACB varies in the literature for vertical fracture configurations. Some authors describe the fracture passing through the sulcus for the tendon of the extensor carpi ulnaris muscle [9,12,32,33], whereas others report it palmar to the sulcus [8,34,35,36]. In the largest case series to date, Barr et al. located the fracture in the midportion of the ACB palmar to the lateral sulcus [8]. Finite element modeling based on compression testing similarly demonstrated peak stress concentrations in a vertical plane between one-half and two-thirds of the bone’s longitudinal extent [37]. To reproduce the most commonly reported configuration and enhance clinical relevance, fractures in the present study were therefore created palmar to the sulcus.

Radiographically, widening of the fracture gap and displacement of the palmar fragment are characteristic findings in naturally occurring vertical plane fractures of the ACB when images are obtained in carpal flexion [34,36]. In the present cadaveric specimens, measurement of fracture gap width showed considerable variability. Interfragmentary distance was therefore assessed descriptively rather than relying solely on absolute measurements.

One specimen did not show fracture gap widening during maximal flexion, likely due to incomplete transection of the fascia overlying the lateral ACB during fracture creation, which preserved residual soft tissue tension and restricted fragment displacement. Medial soft tissue attachments were not specifically transected and may also have con-tributed to residual stability.

A fibrous connection between the ACB and the thicker lateral branch of the metacarpal flexor retinaculum has been described [38]. Because the fractures in this study were experimentally induced, this structure was likely preserved, which may explain the distal displacement of the palmar fragment observed during maximal extension.

In naturally occurring fractures, traumatic disruption of surrounding soft tissue attachments likely reduces these stabilizing forces and facilitates fragment displacement [34,39].

In the neutral carpal position and maximal extension, most specimens demonstrated closure of the fracture gap. This may be explained by compressive forces across the fracture plane generated by the antebrachial fascia and flexor retinaculum during extension, resulting in passive fragment reduction. Similar findings have been reported previously [17,34]. Clinically, fracture gap compression during surgical reduction is facilitated when the limb is maintained in extension [17], allowing more effective application of pointed reduction forceps. The findings of the present study support this observation, as fracture gap closure was predominantly observed in the neutral carpal position and maximal extension. In two specimens, a persistent visible fracture gap was observed in both neutral position and maximal extension, which may be attributed to loss of bone material during fracture creation.

With the increasing availability of standing CT, it should be considered that partial carpal flexion during image acquisition may influence fracture gap configuration and fragment displacement. In recumbent CT performed under general anaesthesia, recovery without prior stabilization may pose a risk depending on the fracture configuration. Based on the consistent morphology of the ACB and the biomechanical findings of the present study, it may be feasible in selected cases, particularly in non-comminuted vertical fractures, to perform CT imaging and surgical repair within the same anaesthetic episode. This approach may allow preoperative planning and implant selection in advance, thereby facilitating more efficient and potentially safer fracture management.

## 5. Conclusions

This study provides the first CT-based morphometric characterization of the ACB and demonstrates that it exhibits independent physiological motion relative to the other carpal bones during carpal flexion. The consistent geometric configuration observed among horses of comparable size suggests that anatomically contoured, size-specific implant designs may be feasible. Position-dependent displacement of the palmar fragment underscores the importance of limb positioning for both diagnostic imaging and surgical management. The findings further indicate that compressive forces within the proposed “nutcracker” configuration alone are unlikely to account for fracture development. Despite its ex vivo design, this study establishes a framework for future investigations into the biomechanical mechanisms underlying ACB fracture formation and supports the design of anatomically adapted implants and refined surgical strategies.

## Figures and Tables

**Table 1:** Interfragmentary distance at different carpal positions. Values represent the number and percentage of specimens. Fracture gaps were classified as visible (open) or closed on MPR CT images. Interobserver agreement is reported as Fleiss’ kappa. Symbols (*, †) indicate interobserver differences.

Position	Fracture Gap Visible n (%)	Fracture Gap Closure n (%)	Fleiss’ Kappa
Neutral	4 (33.3) *	8 (66.7)	0.747
Maximal flexion	11 (91.7) †	1 (8.3)	0.777
Maximal extension	4 (33.3) *	8 (66.7)	0.909

**Table 2:** Palmar fragment displacement according to limb position. Displacement was categorized as absent, proximal, or distal on MPR CT images. Values represent number and percentage of specimens. In cases of equal disagreement between evaluators (2 vs. 2), no definitive classification was assigned and these cases are reported as “no agreement”. Interobserver agreement is reported as Fleiss’ kappa. Symbols (*, †) indicate interobserver differences. Representative CT images are shown in the table header.

Position	No Displacement n (%) 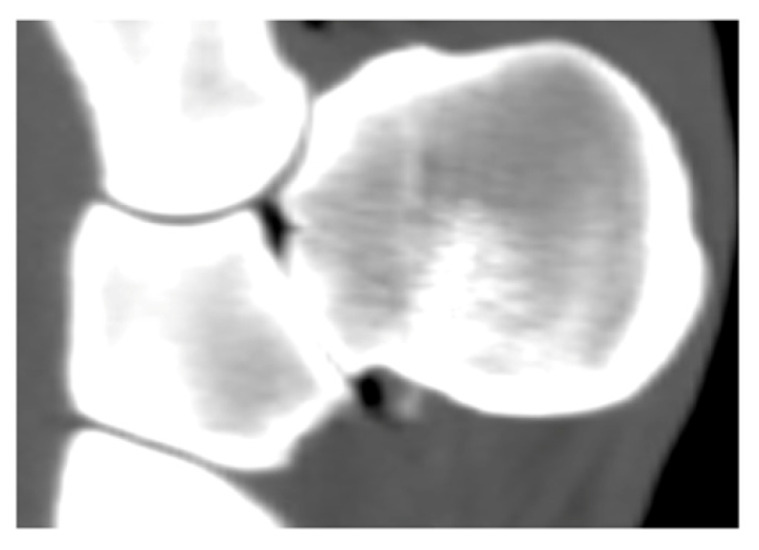	Proximal Displacement n (%) 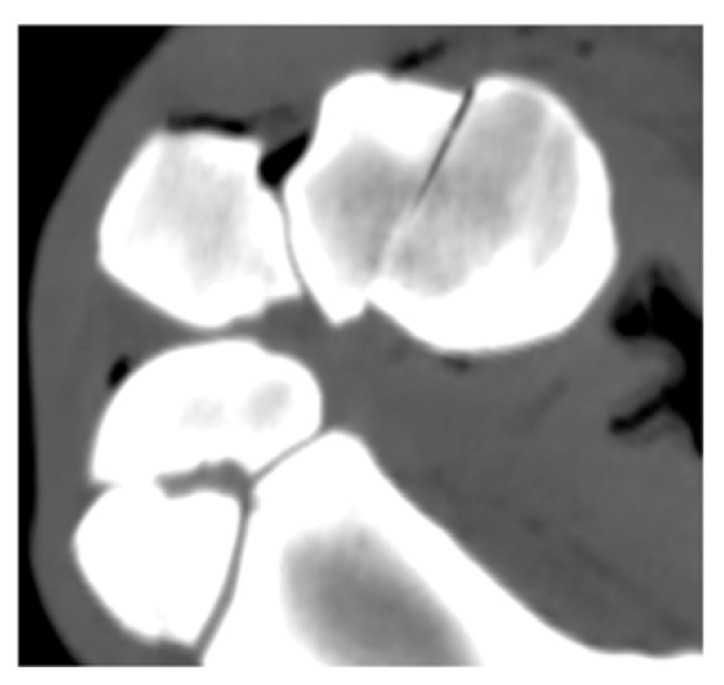	Distal Displacement n (%) 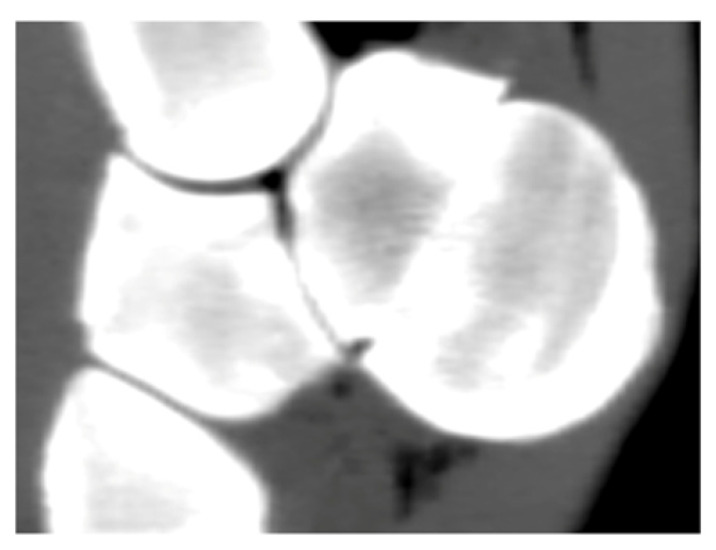	No Agreement n (%)	Fleiss’ Kappa
Neutral	10 (83.3)	0 (0)	2 (16.7)	0 (0)	1.000
Maximal flexion	2 (16.7) †	8 (66.7)	1 (8.3)	1 (8.3)	0.672
Maximal extension	4 (33.3) *	0 (0)	8 (66.7)	0 (0)	0.909

## Data Availability

The original contributions presented in this study are included in the article/Supplementary Material. Further inquiries can be directed to the corresponding author.

## Supplementary Materials

The following supporting information can be downloaded at: https://www.mdpi.com/article/10.3390/ani16081132/s1. Supplementary S1 and S2: Video of three-dimensional computed tomography; Supplementary S3: Bland–Altman results table; Supplementary S4: Summary of morphometric measurements; Supplementary S5: Heatmap of the Pearson correlation matrix; Supplementary S6: Scatterplot matrix; Supplementary S7: Video of CT-volume of specimen in maximal carpal flexion.

## Abbreviations

The following abbreviations are used in this manuscript:

ACB	Accessory carpal bone
CT	Computed tomography
3D CT	Three-dimensional computed tomography
MPRs	Multiplanar reconstructions
