# Correction: Gernhardt et al. Ex Vivo Computed Tomographic Morphometry and Motion of the Native and Fractured Equine Accessory Carpal Bone. *Animals* 2026, *16*, 1132

**DOI:** 10.3390/ani16121866

**Published:** 2026-06-17

**Authors:** Jennifer Gernhardt, Thomas Reuter, Guido Fritsch, Nicole Schulze, Kathrin Mählmann, Christoph Lischer

**Affiliations:** 1 Equine Clinic, Freie Universität Berlin, Oertzenweg 19b, 14163 Berlin, Germany; 2 ICM—Institut Chemnitzer Maschinen- Und Anlagenbau e.V., 09117 Chemnitz, Germany; 3 Leibniz Institute for Zoo and Wildlife Research, Forschungsverbund Berlin e.V., 10315 Berlin, Germany

In the original publication [1], there was a mistake in Figure 1 as published. The corrected Figure 1 appears below. The authors state that the scientific conclusions are unaffected. This correction was approved by the Academic Editor. The original publication has also been updated.

## Figures and Tables

**Figure 1:**
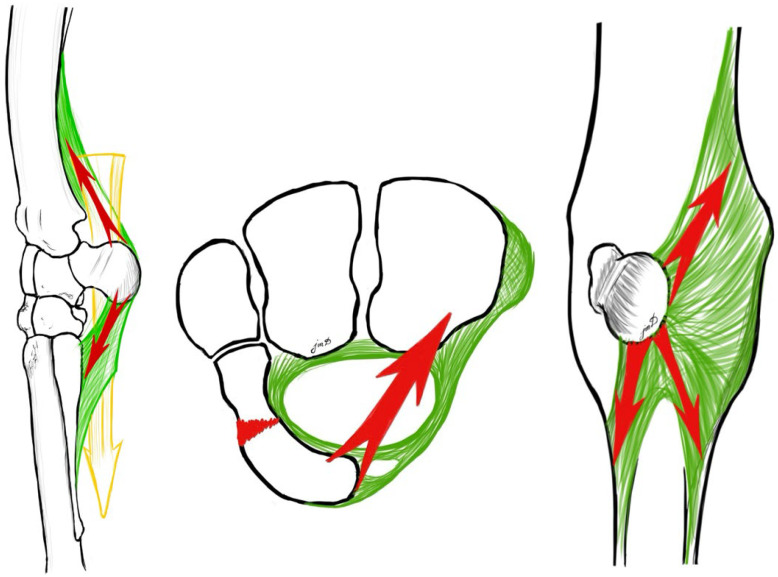
**Left**: Lateral view of the left carpus in hyperextension (dorsal to the left). Illustration by Prof. Jean-Marie Denoix demonstrating opposing tensile forces acting on the accessory carpal bone (ACB). Ligamentous attachments and the antebrachial fascia generate proximally and distally directed traction forces (red arrows). In addition, the flexor tendons within the carpal canal are subjected to increased tension during hyperextension of the limb. **Middle**: Transverse view of the proximal carpal row (dorsal to the top). During hyperextension, the flexor retinaculum is maximally tensioned, exerting compressive force on the palmar aspect of the ACB while simultaneously applying a medially directed force (red arrow). **Right**: Tensile forces exerted by the antebrachial fascia (red arrows) in distal and proximomedial directions [16].
