# Supplementary material for: Ex Vivo Computed Tomographic Morphometry and Motion of the Native and Fractured Equine Accessory Carpal Bone
Source: Animals (Basel). 2026 Apr 8;16(8):1132. doi: 10.3390/ani16081132 (PMC13113565; doi:10.3390/ani16081132)
Supplement: Supplementary file 1 [file animals-16-01132-s001.zip › Supplementary 3.pdf]

Parameter	Comparison Measurement		Bias	Lower LoA	Upper LoA	Valid pairs (n)
DPW-R	1	2	0.000	-0.061	0.062	20
DPW-R	1	3	0.007	-0.049	0.062	20
DPW-R	2	3	0.006	-0.025	0.038	20
LMT-DM	1	2	-0.008	-0.152	0.137	20
LMT-DM	1	3	-0.004	-0.085	0.077	20
LMT-DM	2	3	0.004	-0.147	0.155	20
LMT-DS	1	2	0.001	-0.038	0.039	20
LMT-DS	1	3	0.001	-0.040	0.043	20
LMT-DS	2	3	0.001	-0.033	0.035	20
LMT-PS	1	2	0.001	-0.046	0.048	20
LMT-PS	1	3	-0.001	-0.036	0.033	20
LMT-PS	2	3	-0.003	-0.039	0.033	20
DPW-U	1	2	-0.011	-0.045	0.023	20
DPW-U	1	3	-0.008	-0.062	0.046	20
DPW-U	2	3	0.003	-0.040	0.047	20
LMT-DC	1	2	0.002	-0.038	0.042	20
LMT-DC	1	3	0.003	-0.044	0.050	20
LMT-DC	2	3	0.001	-0.048	0.050	20
LMT-DPC	1	2	-0.003	-0.038	0.033	20
LMT-DPC	1	3	0.002	-0.033	0.037	20
LMT-DPC	2	3	0.005	-0.028	0.037	20
LMT-PC	1	2	-0.007	-0.064	0.051	20
LMT-PC	1	3	-0.003	-0.061	0.054	20
LMT-PC	2	3	0.003	-0.043	0.050	20
PDL-C	1	2	0.001	-0.045	0.047	20
PDL-C	1	3	0.004	-0.042	0.050	20
PDL-C	2	3	0.003	-0.021	0.027	20
DRM	1	2	0.001	-0.037	0.038	20
DRM	1	3	-0.006	-0.036	0.024	20
DRM	2	3	-0.007	-0.045	0.032	20
PRM	1	2	-0.004	-0.023	0.015	20
PRM	1	3	-0.018	-0.164	0.129	20
PRM	2	3	-0.014	-0.159	0.132	20
DRL	1	2	0.008	-0.050	0.065	20
DRL	1	3	0.008	-0.056	0.072	20
DRL	2	3	0.000	-0.013	0.014	20
PRL	1	2	-0.002	-0.026	0.021	20
PRL	1	3	-0.001	-0.021	0.018	20
PRL	2	3	0.001	-0.014	0.016	20
MAXFLEX	1	2	-0.219	-1.873	1.435	12
MAXFLEX	1	3	-0.422	-2.546	1.702	12
MAXFLEX	2	3	-0.203	-2.507	2.100	12
MAXEXT	1	2	0.237	-1.628	2.103	11
MAXEXT	1	3	0.115	-1.615	1.845	11
MAXEXT	2	3	-0.122	-1.569	1.325	11

Bias represents the mean difference between paired measurements. Limits of agreement (LoA) were calculated as the mean difference ± 1.96 standard deviations. Valid pairs (n) indicate the number of non-missing paired observations included in each comparison. All analyses were performed using pairwise complete observations.
