# Supplementary material for: Ex Vivo Computed Tomographic Morphometry and Motion of the Native and Fractured Equine Accessory Carpal Bone
Source: Animals (Basel). 2026 Apr 8;16(8):1132. doi: 10.3390/ani16081132 (PMC13113565; doi:10.3390/ani16081132)
Supplement: Supplementary file 1 [file animals-16-01132-s001.zip › Supplementary 5.pdf]

	DPW-R	PDL-C	DPW-U	LMT-DM	LMT-DS	LMT-PS	LMT-DC	LMT-DPC	LMT-PC	PRM	DRM	PRL	DRL	MAXEXT	MAXFLEX	BW	A
DPW-R	1	0.00119	-0.01784	-0.16195	0.04423	0.20202	-0.17763	-0.41691	**-0.44878**	**0.66641**	**0.68497**	**0.67141**	**0.67036**	-0.24341	-0.22419	0.17983	**-0.68218**
PDL-C	0.00119	1	**0.78701**	0.0237	0.17239	0.03687	**0.76784**	**0.71061**	**0.69102**	0.20641	0.21484	0.3472	0.35671	0.17442	0.02017	**0.45089**	0.12474
DPW-U	-0.01784	**0.78701**	1	0.17169	0.28858	0.15022	**0.84352**	**0.77107**	**0.73405**	0.32803	0.34858	**0.48566**	**0.49728**	0.18139	0.1531	0.25176	0.00209
LMT-DM	-0.16195	0.0237	0.17169	1	**0.649**	0.23964	0.22807	0.29177	0.13115	-0.30482	-0.2998	-0.21877	-0.21026	0.50358	0.34917	-0.15225	0.11278
LMT-DS	0.04423	0.17239	0.28858	**0.649**	1	**0.71838**	**0.46753**	0.42077	0.18496	-0.20276	-0.18858	-0.03895	-0.0243	0.21782	0.09376	0.02276	-0.10677
LMT-PS	0.20202	0.03687	0.15022	0.23964	**0.71838**	1	0.32504	0.0714	-0.008	0.01072	0.02595	0.09774	0.10813	0.12767	0.27281	-0.1401	-0.26695
LMT-DC	-0.17763	**0.76784**	**0.84352**	0.22807	**0.46753**	0.32504	1	**0.83579**	**0.70804**	0.08337	0.08671	0.2332	0.24295	0.39687	0.10746	0.32304	0.12515
LMT-DPC	-0.41691	**0.71061**	**0.77107**	0.29177	0.42077	0.0714	**0.83579**	1	**0.8019**	-0.18617	-0.17432	-0.00997	0.00262	0.20039	0.0346	0.26515	0.22965
LMT-PC	**-0.44878**	**0.69102**	**0.73405**	0.13115	0.18496	-0.008	**0.70804**	**0.8019**	1	-0.15626	-0.15704	-0.00767	-8.06E-04	0.18483	0.06047	**0.43998**	**0.45778**
PRM	**0.66641**	0.20641	0.32803	-0.30482	-0.20276	0.01072	0.08337	-0.18617	-0.15626	1	**0.9958**	**0.96814**	**0.9644**	-0.40593	-0.10538	-0.01635	**-0.65219**
DRM	**0.68497**	0.21484	0.34858	-0.2998	-0.18858	0.02595	0.08671	-0.17432	-0.15704	**0.9958**	1	**0.97042**	**0.96821**	-0.42073	-0.12418	-0.0104	**-0.65517**
PRL	**0.67141**	0.3472	**0.48566**	-0.21877	-0.03895	0.09774	0.2332	-0.00997	-0.00767	**0.96814**	**0.97042**	1	**0.99959**	-0.3628	-0.08178	0.06494	**-0.65264**
DRL	**0.67036**	0.35671	**0.49728**	-0.21026	-0.0243	0.10813	0.24295	0.00262	-8.06E-04	**0.9644**	**0.96821**	**0.99959**	1	-0.36764	-0.0908	0.06692	**-0.65053**
MAXEXT	-0.24341	0.17442	0.18139	0.50358	0.21782	0.12767	0.39687	0.20039	0.18483	-0.40593	-0.42073	-0.3628	-0.36764	1	0.52128	**0.04666**	0.23223
MAXFLEX	-0.22419	0.02017	0.1531	0.34917	0.09376	0.27281	0.10746	0.0346	0.06047	-0.10538	-0.12418	-0.08178	-0.0908	0.52128	1	-0.53808	0.12521
BW	0.17983	**0.45089**	0.25176	-0.15225	0.02276	-0.1401	0.32304	0.26515	**0.43998**	-0.01635	-0.0104	0.06494	0.06692	0.04666	-0.53808	1	0.2
A	**-0.68218**	0.12474	0.00209	0.11278	-0.10677	-0.26695	0.12515	0.22965	**0.45778**	**-0.65219**	**-0.65517**	**-0.65264**	**-0.65053**	0.23223	0.12521	0.2	1

1
0.8
0.6
0.4
0.2
0
-0.2
-0.4
-0.6
-0.8
-1

Bold black significant correlations according to Pearson -1 < r < 1, p < 0.05 (A - Age, BW - Body Weight)

Supplementary Figure 5. Heatmap of the Pearson correlation matrix for the analyzed variables (n = 20 per variable, except for angle measurements, n = 12 and n = 11). Correlations were calculated using the mean values of three measurements per variable. Color intensity represents the strength and direction of the linear relationships (blue = positive, red = negative). Correlations significant at p < 0.05 are indicated in bold black.

Abbreviations:
DPW-R, dorsopalmar width at point R;
DPW-U, dorsopalmar width at point U;
PDL-C, proximodistal length at point C;
LMT, lateromedial thickness;
DM, dorsal margin of the sulcus;
DS, deepest point of the sulcus;
PM, palmar margin of the sulcus;

DC, dorsal margin of the medial concavity;
DPC, deepest point of the medial concavity;
PC, palmar margin of the medial concavity;
DRM, dorsal radius of medial concavity;
PRM, palmar radius of medial concavity;
DRL, dorsal radius of lateral convexity;
PRL, palmar radius of lateral convexity;
MAXEXT, maximal extension;
MAXFLEX, maximal flexion;
BW, body weight;
A, age.
