# Supplementary material for: Ex Vivo Computed Tomographic Morphometry and Motion of the Native and Fractured Equine Accessory Carpal Bone
Source: Animals (Basel). 2026 Apr 8;16(8):1132. doi: 10.3390/ani16081132 (PMC13113565; doi:10.3390/ani16081132)
Supplement: Supplementary file 1 [file animals-16-01132-s001.zip › Supplementary 6.pdf]

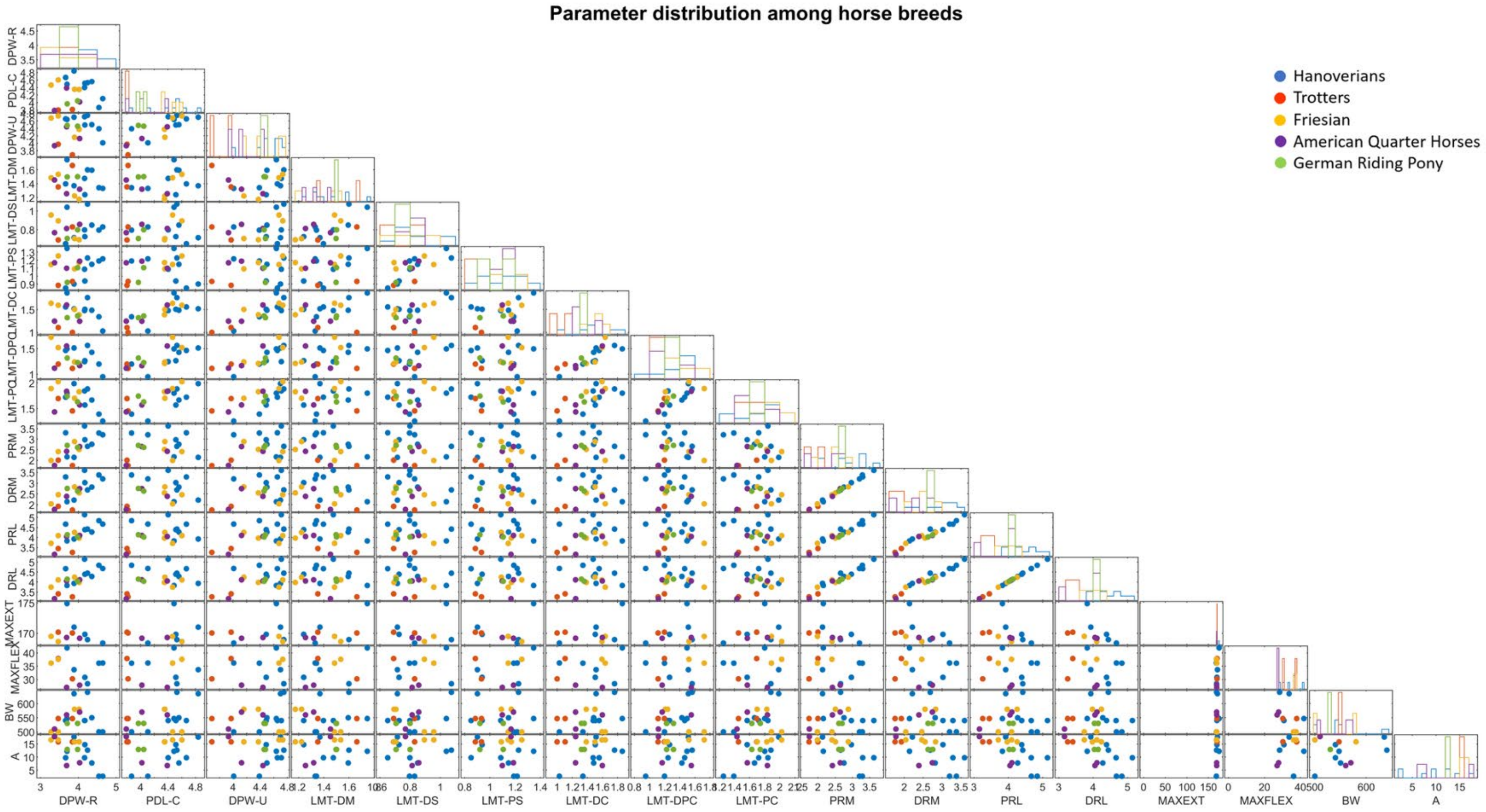

Supplementary Figure 6. Scatterplot matrix illustrating pairwise relationships among the analyzed variables (n = 20 per variable, except for angle measurements, n = 12 and n = 11). Diagonal panels show the distribution of each variable, and off-diagonal panels show scatterplots. Colors indicate horse breed.

Abbreviations:

DPW-R, dorsopalmar width at point R;

DPW-U, dorsopalmar width at point U;
PDL-C, proximodistal length at point C;
LMT, lateromedial thickness;
DM, dorsal margin of the sulcus;
DS, deepest point of the sulcus;
PM, palmar margin of the sulcus;
DC, dorsal margin of the medial concavity;
DPC, deepest point of the medial concavity;
PC, palmar margin of the medial concavity;
DRM, dorsal radius of medial concavity;
PRM, palmar radius of medial concavity;
DRL, dorsal radius of lateral convexity;
PRL, palmar radius of lateral convexity;
MAXEXT, maximal extension;
MAXFLEX, maximal flexion;
BW, body weight;
A, age.
